# Bird Community Conservation and Carbon Offsets in Western North America

**DOI:** 10.1371/journal.pone.0099292

**Published:** 2014-06-11

**Authors:** Richard Schuster, Tara G. Martin, Peter Arcese

**Affiliations:** 1 Department of Forest and Conservation Sciences, University of British Columbia, Vancouver, British Columbia, Canada; 2 Ecosciences Precinct, CSIRO Ecosystem Sciences, Brisbane, Queensland, Australia; University of Alberta, Canada

## Abstract

Conservation initiatives to protect and restore valued species and communities in human-dominated landscapes face huge challenges linked to the cost of acquiring habitat. We ask how the sale of forest carbon offsets could reduce land acquisition costs, and how the alternate goals of maximizing α or β-diversity in focal communities could affect the prioritization land parcels over a range of conservation targets. Maximizing total carbon storage and carbon sequestration potential reduced land acquisition costs by up to 48%. Maximizing β rather than α-diversity within forest and savannah bird communities reduced acquisition costs by up to 15%, and when these solutions included potential carbon credit revenues, acquisition cost reductions up to 32% were achieved. However, the total cost of conservation networks increased exponentially as area targets increased in all scenarios. Our results indicate that carbon credit sales have the potential to enhance conservation outcomes in human-dominated landscapes by reducing the net acquisition costs of land conservation in old and maturing forests essential for the persistence of old forest plant and animal communities. Maximizing β versus α-diversity may further reduce costs by reducing the total area required to meet conservation targets and enhancing landscape heterogeneity. Although the potential value of carbon credit sales declined as a fraction of total acquisition costs, even conservative scenarios using a carbon credit value of $12.5/T suggest reductions in acquisition cost of up to $235 M, indicating that carbon credit sales could substantially reduce the costs of conservation.

## Introduction

There is a pressing need to develop mechanisms to promote biodiversity conservation in the face of climate and land use change and the competing needs of humans [1]–[4]. This challenge is particularly severe in human-dominated landscapes, where private ownership prevails and the cost of purchasing properties or compensating land holders for lost opportunity incurred as a result of conservation can be substantial [5], [6]. There are multiple routes available to land conservation, such as land purchase or private land conservation initiatives [7]–[10]. Here we focus on land purchase rather than conservation agreements on private lands, to avoid having to estimate the long-term costs of monitoring and enforcement or the probability that private conservation agreements are challenged in future [7], [11], [12]. One way of making conservation via land purchase more affordable is by offsetting those costs via payments for ecosystem services. The use of carbon markets to pay for carbon sequestration is an ecosystem service gaining global attention [13]–[15], in part because public concerns about the consequences of climate change have motivated 35 nations and 13 sub-national jurisdictions to put a price on carbon [16]. To the degree that carbon and biodiversity values overlap, carbon offsets could therefore be used to protect forests that would otherwise be logged [17], [18] or to restore those still supporting valued old forest communities [19].

Several outstanding issues arise when considering the role of carbon markets in forest restoration. One issue is that biodiversity values may be lower in stands with the highest returns from carbon sequestration sales because sequestration rates typically peak in stands of intermediate age [20]. In contrast, older forests act as carbon sinks and continue to accumulate carbon over time, but at lower rates on average [21], resulting in high initial returns on the sale of carbon storage credits, where one carbon credit represents the offset of greenhouse gas emissions by one tonne of carbon dioxide equivalent (CO_2_-e). A second issue is whether to develop conservation plans that maximize species richness (α-diversity) within habitats or maximize dissimilarities in community composition (β-diversity) to accommodate landscape complexity and species that utilize multiple habitats [22], [23]. Under climate change, it has been suggested that an emphasis on community dissimilarity (β-diversity) may deliver more robust conservation plans than those based on species richness [23], [24]. Here we examine the potential value of carbon credit sales to offset land acquisition costs by developing conservation area designs that maximize β or α-diversity in native old forest and savannah bird communities in relation to forest structure and human land use. Specifically, we ask how protecting forests with high carbon storage versus high carbon uptake is likely to affect conservation outcomes.

### Carbon and Biodiversity in the Georgia Basin

The Coastal Douglas Fir (CDF) ecozone of the Georgia Basin (British Columbia, Canada [25]) is a classic example of an endangered but extraordinarily diverse region that has been rapidly converted to exclusive human use (≥60%) [26] and thus retains ≤0.3% of historic old forests (>250 years) [27] and ≤10% of oak woodland and savannah [28], both of which provide habitat for 117 species at risk of extirpation, which represents the highest density of species of global and provincial concern to conservation of any ecozone in BC [26]. Because regional, provincial and federal authorities own <20% of the region and only ∼9% is already conserved, cost-efficient routes to conservation are urgently needed to help reduce the risk of extirpation for those species and related ecosystems.

Prior to European colonization the CDF occurred as uneven-aged forest (often >300 years) dissected by shallow and deep-soil meadow and woodland communities [25], [29] maintained in part by aboriginal land management practices to enhance hunting opportunities and root and fruit harvests [30]–[33]. In addition to recent human-caused disturbances, oak woodland and savannah community distributions are predicted to shift under future climate conditions, and only a small fraction of the current protected areas have the potential to accommodate this shift [34]. The resulting land use heterogeneity within the region and potential for humans to directly or indirectly affect native species richness [19], [35]–[37] make this system ideal for studying trade-offs involved when attempting to maximize α- versus β-diversity in conservation plans, while simultaneously maximizing ecosystem service values represented as total carbon stored or sequestration potential. To do so, we compared systematic conservation scenarios that maximized old forest and savannah bird biodiversity (α-diversity) or their dissimilarity (β-diversity), and then quantified their relative costs given alternate carbon markets, and in relation to increasing targets for the total area conserved [38] (Table 1).

**Table 1:** Summary of diversity features, land cost metrics, conservation targets and carbon prices used in 144 Marxan scenarios.

Diversity features (n = 2)	Property cost metrics (n = 4)	Conservation Targets [%] (n = 9)	Carbon credit value (CC) (n = 3)
α-diversity (maximize Old Forest + Savannah individually)	Total Land value (TLV)	10 to 50 (in 5% steps)	9 $/T (lowest price PCT has paid for credits so far)
β-diversity (maximize β-score)	TLV – StC	-	12.5 $/T (half the cost PCT charges, as well as roughly the average price PCT is paying for credits)
	TLV – SeqC		25 $/T (the price that PCT is charging for credits)
	TLV – TotC		

PCT =  Pacific Carbon Trust; StC =  Carbon Storage * CC; SeqC =  Carbon.

Sequestration potential * CC; TotC = StC+SeqC.

## Materials and Methods

### Ethics Statement

Permits or permission for the use of bird point count locations were obtained from Parks Canada (locations in National Park Reserves), private land owners (locations on private land), or did not require specific permission as they occurred on public right of ways (e.g., roadsides, regional parks). As private land owners did not want their information posted publically please contact the authors for contact details. The field studies did not involve endangered or protected species. This study did not require approval from an Animal Care and Use Committee because it was a non-invasive observational field study, and did not involve the capture and handling of wild animals.

### Biodiversity data

**Figure 1:**
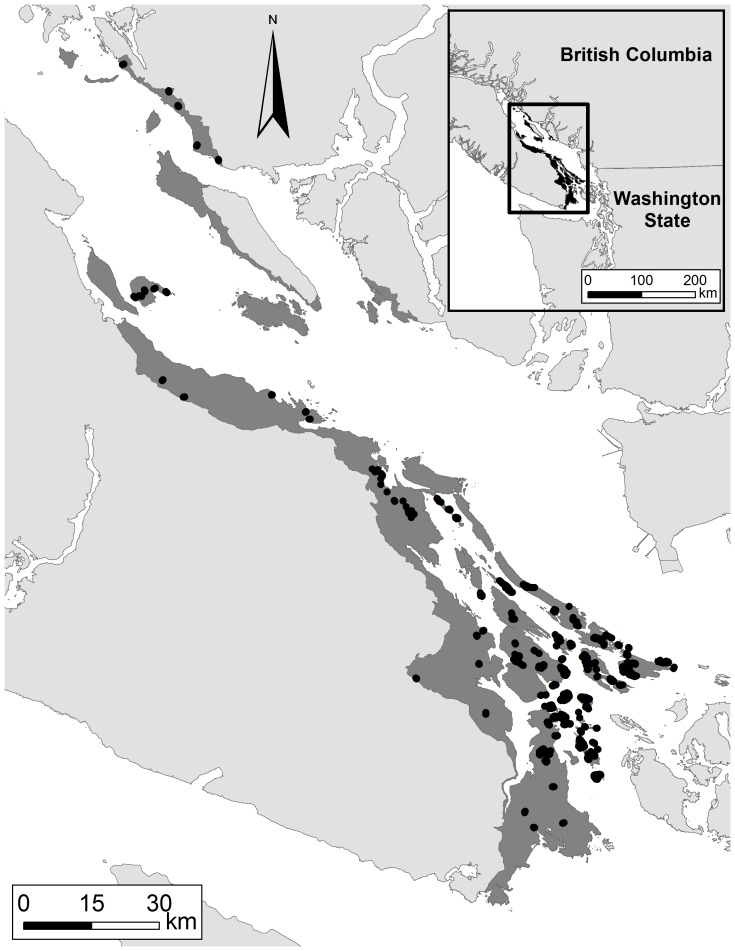
Georgia Basin study area including bird point count locations. Dark grey area indicates the extent of the study region and black dots represent bird point count locations.

We used trained observers to conduct 1,770 point counts on mainland BC and 53 islands from 30 Apr–11 Jul, 2005–2011 (Fig.1, 48.7° N, 123.5° W) to record all birds detected in 10 min, 50 m radius counts between 5 AM–12 PM at 713 sample locations (>100 m apart). Locations were re-visited 1–12 times and geo-referenced via a GPS (GPS60, Garmin Ltd, Kansas, USA). We extended the approach of Schuster & Arcese [19] geographically (from 1560 km^2^ to 2520 km^2^) by adding 601 counts to create predictive distribution models for 47 bird species and 25 covariates based on remote-sensed data and models incorporating imperfect detectability [39]. To estimate detectability we used one site specific (crown closure) and three observation specific (time of date, Julian date and observer identity) covariates.

We associated bird species indicators with the habitats they were expected to occupy by using 11 experts to rank the likelihood of observing 47 species in 10 focal habitat types using photographic and text descriptions of herbaceous, shrub, woodland, wetland, four forest types (pole, young, mature and old), and 2 human-dominated habitats (rural, urban), to create two community metrics indicating Old Forest (OF, [19]) and Savannah (SAV) habitats standardized between 0 and 1 by dividing through the maximum value possible (details in Appendix S1), where:
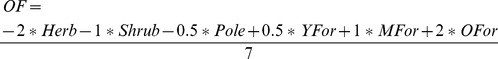


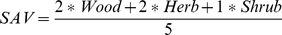



These metrics match our goals given the region's history and focus on Old Forest and Savannah community conservation (see Introduction). Specifically, each species contributed to the cumulative Old Forest or Savannah community score, weighted by its expert opinion score for the given sub-type, summed across species to create community specific association scores from 0 to 1, and corresponding to none versus all members of the community expected to be present. The metrics were then projected spatially as predictive maps of community occurrence over the entire study area (2520 km^2^, Fig. 1) as 1 ha hexagonal polygons (Fig. S1–S2 in Appendix S1,see also [19]).

### Carbon estimates

Forest carbon storage and sequestration rates were estimated for all forested land in the study area using terrestrial ecosystem mapping (TEM; [27]) and FORECAST [40]. FORECAST is a stand-level forest ecosystem simulator that is one of two models approved by the BC Ministry of Forests for carbon budget assessments [41], and the only model calibrated for use in the CDF (Blanco et al. 2007) and linked to TEM [42]. To facilitate carbon analysis TEM polygons were stratified into homogenous analysis units based on site series. Net ecosystem carbon storage was limited to: above and below-ground tree biomass, deadwood biomass, and dead below-ground biomass. Each analysis unit was simulated for a period of 300 years with results reported for annual time steps to create carbon storage curves. FORECAST results were subsequently assigned to individual TEM polygons by estimating the age of each polygon subsection based upon the current assigned structural stage and estimated productivity class [42]. These age estimates were derived from ranges provided by Meidinger *et al.* [43] for regional forest ecosystems. Ages of old stands (structural stage 7) were set at 200 to be conservative. Age estimates were verified against a subset of TEM polygons (Southern Gulf Islands of Southwestern BC) for which direct age estimates were available (n = 254). For conservation prioritization analysis we used predicted net ecosystem carbon storage and net ecosystem carbon sequestration estimates for 20 years from now due to uncertainty about fire frequency in the future. Further details on this analysis are provided in Appendix S2.

### Cadastral layer and property costs

We incorporated spatial heterogeneity in land values [5], [44]–[46] in our plans by using cadastral data and 2012 land value assessments (Integrated Cadastral Information Society of BC, ICIS). However, because there is no centralized entity curating cadastral data for British Columbia, we combined data from ICIS, the BC Assessment agency and the Integrated Cadastral Fabric. Doing so required processing to remove stacked and overlapping polygons and slivers. The combined cadastral layer included 193,623 polygons. Current assessments were available for 187,139 polygons, but missing for 3,281 polygons or reduced relative to market value due to taxation or administrative reasons unrelated to our work (e.g farm or managed forest land, 3,203 ploygons). For these 6,484 polygons we applied an inverse distance weighted interpolation to estimate land values by splitting cadastral polygons into 10 groups based on polygon size to accommodate high size related heterogeneity in assessed cost using R v.2.15.2 [47] and packages gstat v.1.0-14 [48] and sp v.1.0-1 [49].

We used tax assessment land values to estimate acquisition costs because they are revised annually in the region, and because more ‘realistic’ strategies would require speculation on how purchase cost may be affected by location of existing reserves, evolving zoning plans, the willingness of owners to sell, or other effects. In particular, there is as yet no consensus on the effect of conservation agreements on land values [50]–[52].

### Marxan inputs

We used Marxan [53] to prioritize cadastral polygons for inclusion in conservation area designs by using them as planning units (n = 193,623). We calculated biodiversity and carbon estimates for each planning unit using ArcGIS v.10.1 [54] and area weighted sums in Geospatial Modelling Environment v.0.7.2.1 [55].

To determine whether maximizing β- versus α- diversity affected conservation outcomes we created two sets of diversity features as inputs to Marxan. First we included the diversity features individually in the analysis and set conservation targets for Old Forest and Savannah scores as the percentage of total old forest or savannah habitat existing within the study region. The second approach we used was to pre-specify a β-diversity metric to combine biodiversity features with the goal to specifically maximize highly diverse habitat patches. For this purpose we created the following metric:
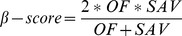



This represents the Old Forest and Savannah community dissimilarity, using a scaling factor of 2 to create β-scores between 0 and 1 (Fig. S3 in Appendix S1). In Marxan analyses we set targets for the β-score, while still including Old Forest and Savannah metrics (without setting a target) to keep track of individual community representation. We used a total of four property cost metrics per diversity scenario: i) Total land value (TLV) for each property, which is the sum of the assessed property value and any improvement on that parcel; ii) TLV minus the current carbon storage (T) times the carbon credit value ($/T). Here we used $12.5 Canadian per credit, which is half the amount that Pacific Carbon Trust, a crown corporation established in 2008 to deliver greenhouse gas offsets in the province of British Columbia (http://pacificcarbontrust.com, date accessed: 2013-12-10), sells credits for and about the average amount they pay for credits. iii) TLV minus the amount of potential carbon sequestration over 20 years times the carbon credit value; iv) TLV minus ii and iii combined (Table 1).

### Marxan scenarios

We used the two diversity scenarios α (Old Forest + Savannah) and β (β-score) in combination with the four cost scenarios (Table 1). An important consideration for this study was what level to set the required conservation target to, in order to ensure the study system will maintain viable populations of native species and be resilient to predicted environmental change in the future. As there is debate about what constitutes appropriate conservation goals [38] we used a range of conservation targets (10–50%) to investigate the potential trade-offs of different targets. We calibrated each diversity scenario to ensure robust analysis by initially setting the diversity target to 50% (the most costly to reach) and the number of restarts to 100, as we were not so much interested in the spatial representation of the reserve design but rather its cost effectiveness [56]. For the same reason we also refrained from setting boundary length modifiers. For each diversity scenario we created Marxan solutions for combinations of the following species penalty factors (SPF's): 1–10,15,20 and number of iterations: 10 k, 50 k, 100 k, 500 k, 1 M, 5 M, 10 M, 25 M, 50 M, 100 M, for a total of 65 calibration analyses per diversity scenario. We created cumulative distribution functions using number of solutions on the y-axis, solution cost on the x-axis for SPF and Marxan score for number of iterations [56]. Based on the results we used the following values for SPF and number of iterations respectively: Old Forest + Svannah (3/10 M); β-score (3/10 M). We also investigated summed solutions to make sure every restart met its targets, excluding ones that missed the target by >5%. For ease of computation we created an R function to batch run Marxan (Appendix S3).

We held the calibrated values constant in subsequent analyses and ran Marxan scenarios for the two diversity metrics in combination with the four cost metrics, using the baseline carbon credit value of $12.5. For each combination we further varied the conservation target from 10–50%. From each run we recorded the cost of the total reserve system averaged over the number or restart (100), while ensuring conservation targets were met. To examine the amount of remaining Old Forest and Savannah communities protected by maximizing β-diversity we compiled community scores as Marxan features in these scenarios without setting targets, allowing us to keep track of Old Forest and Savannah representation without affecting the analysis. We used the results from these analyses to compare the reserve prices within each diversity scenario as well as across scenarios. In addition we calculated the potential cost savings between fee simple acquisition scenarios (TLV only) and ones that utilize the sale of carbon credits. As market prices of carbon credits are highly variable we extended our approach to include variation in carbon credit value, by repeating the entire analysis for two additional carbon credit values: i) $9 per credit (the lowest rate PCT has ever paid for credits), and ii) $25 per credit (the price PCT sells credits for). In total 144 Marxan scenarios were investigated (Table 1). All results presented here relate to the baseline carbon credit value of $12.5 unless otherwise stated.

## Results

### Land acquisition cost, diversity and planning goals

**Figure 2:**
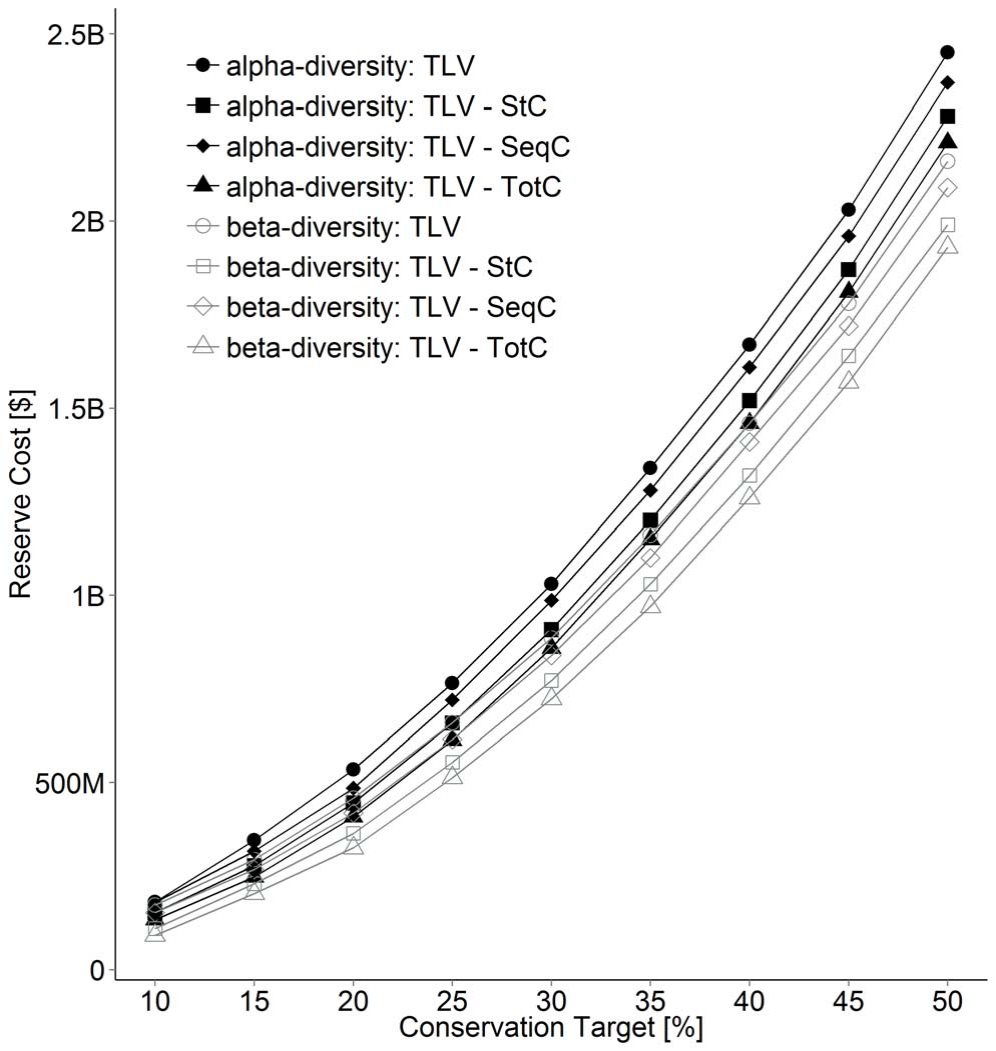
Reserve costs using alpha and beta diversity and a carbon credit value of $12.5 across a range of conservation targets (term definitions in Table 1).

**Figure 3:**
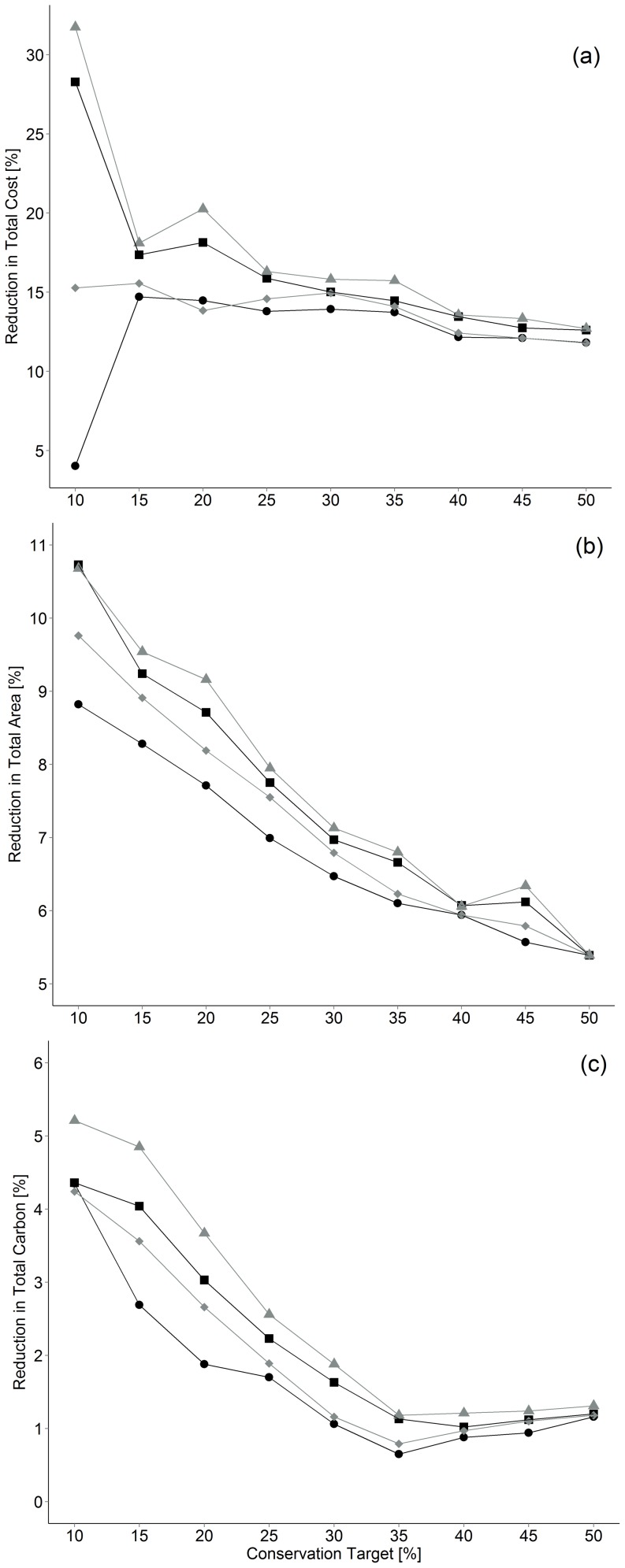
Comparison of α and β-diversity scenario results. Presented are the % reductions when using a β-diversity approach for: a) reserve network cost, b) reserve network area, c) total carbon included in the reserve networks. Circles represent TLV, squares StC, diamonds SeqC and triangles TotC (term definitions in Table 1) (c).

Acquisition costs of conservation networks increased from $180 M to $2.45 B as targets increased from 10 to 50% of remaining Old Forest and Savannah bird communities when maximising α-diversity (Fig. 2), but reduced slightly when maximizing β-diversity ($172 M to $2.16 B, 10–50% target; Fig. 2), representing savings of 4–15% as compared to equivalent α-diversity scenarios depending on conservation target (Fig. 3a). Savings were due in part to a reduction in total area needed to reach a given target when maximising β versus α-diversity (mean  = 7%, range  = 5–11%; Fig. 3b). The amount of standing and sequestration potential carbon in conserved landscapes also declined slightly when maximizing β-diversity (mean = 2%, range = 0.7–5%; Fig. 3c). In contrast, representation of Old Forest communities slightly increased (1–2.5%) and representation of Savannah declined (−2.0–−5.7%) when maximizing β versus α-diversity (Table 2).

**Table 2:** Conservation targets (bold) and % actually included in β-diversity scenarios (BETA) using carbon offset value of $12.5/T.

		Percent of Target Bird Community Protected [%]								
Scenario		10	15	20	25	30	35	40	45	50
Total Land Value (TLV)	BETA	10.00	15.00	20.00	25.00	30.00	35.00	40.00	45.00	50.00
	OF	11.12	16.31	21.60	26.81	32.07	37.01	42.18	47.29	52.41
	SAV	8.80	13.57	18.09	22.87	27.59	32.49	37.22	42.07	46.85
TLV - StC	BETA	10.00	15.00	20.00	25.00	30.00	35.00	40.00	45.00	50.00
	OF	11.18	16.40	21.73	26.89	32.07	37.13	42.23	47.51	52.47
	SAV	8.72	13.38	17.93	22.68	27.45	32.34	37.14	41.78	46.78
TLV - SeqC	BETA	10.00	15.00	20.00	25.00	30.00	35.00	40.00	45.00	50.00
	OF	10.98	16.31	21.67	26.83	32.04	37.10	42.14	47.35	52.43
	SAV	8.96	13.56	18.07	22.85	27.57	32.42	37.30	42.02	46.84
TLV - TotC	BETA	10.00	15.00	20.00	25.00	30.00	35.00	40.00	45.00	50.00
	OF	11.17	16.43	21.72	26.95	31.95	37.18	42.27	47.50	52.48
	SAV	8.72	13.36	17.94	22.70	27.63	32.26	37.10	41.79	46.73

β-targets were met in each case and Old Forest (OF) was generally over and Savannah (SAV) underrepresented. StC =  carbon storage credits; SeqC =  sequestration potential credits; TotC = StC+SeqC.

### Cost savings given carbon credits

Maximizing total (standing + sequestered) carbon resulted in the largest cost savings in both α and β-diversity scenarios aimed at protecting Old Forest and Savannah habitats. Acquisition costs increased from $133 M to $2.21 B as target increased from 10 to 50% when maximizing α-diversity, which represent potentials offset of $47 M–235 M, equivalent to a 10–28% cost reduction via carbon credit sales (Fig.2). In comparison, acquisition costs were lower for scenarios that maximised β-diversity ($90 M to $1.93 B), in part because implied carbon credit sales ($82–227 M) contributed slightly more to cost reduction (e.g., 11–48%; Fig. 2). Maximising carbon storage and carbon sequestration potential individually reduced acquisition costs to a smaller extent, but carbon storage offered superior savings (Fig. 2). Overall, maximizing total carbon returned networks that were 17.5% cheaper on average when maximizing β versus α-diversity compared to 12.3% without using carbon storage and sequestration values (Fig. 3a).

### Conservation targets and carbon price

**Figure 4:**
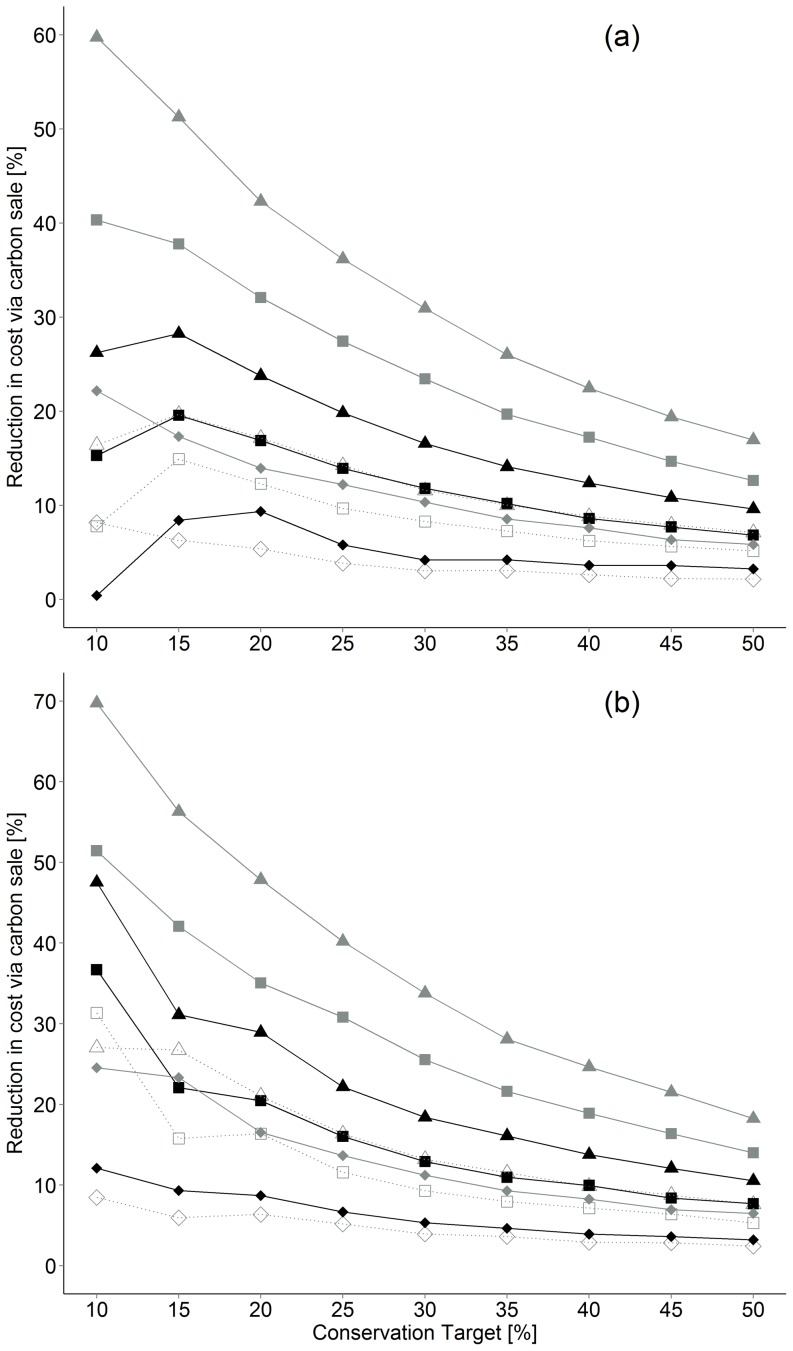
Cost savings when using carbon credit sales in relation to outright acquisition (TLV scenarios). Carbon credit values used are shown in parenthesis for a) α-diversity and b) β-diversity. Rectangles represent StC, diamonds SeqC and triangles TotC. Carbon credit values are as follows: dotted ($9), black ($12) and grey ($25) (term definitions in Table 1).

The cost of conservation networks increased exponentially with increasing targets for all scenarios (Fig. 2). In β-diversity scenarios the total area that needed to be acquired to reach a conservation target was 11–5% lower and acquisition costs 32–13% less than scenarios that maximized α -diversity (Fig. 3). The percent reduction in total acquisition costs due to carbon value also declined as conservation targets increased in α and β-diversity scenarios (Fig. 4a,b). The magnitude by which acquisition costs were reduced by carbon value was similar across prices considered but maximized at $25/T in most scenarios (Fig. 4a,b). Relative reduction in cost due to carbon was maximized at the 10% target in all β-diversity scenarios (Fig. 4b).

## Discussion

Carbon credit sales have the potential to reduce land acquisition costs by up to 48% in Coastal Douglas fir forest and woodland communities of western North America given values already paid in the region ($12.5/T: [57]; Fig. 4b). The largest benefits were realized in scenarios that maximized total carbon and β-diversity in native woodland and old forest bird communities of the region, because those scenarios achieved their targets by selecting cheaper and slightly smaller networks than scenarios maximizing α-diversity in these communities independently. We now develop these points in light of literature on ecosystem services, land acquisition and conservation applied to threatened plant and animal communities in human-dominated landscapes of the Georgia Basin of western North America.

### Maximizing β versus α diversity

Prioritizing β over α-diversity in Old Forest and Savannah bird communities reduced acquisition costs by up to 15%, or up to 32% including carbon values (Fig.3a). One reason for these savings is that the fraction of Old Forest bird habitat included in conservation networks was larger in β as compared to α-diversity scenarios (Table 2), resulting in more carbon stored per unit area conserved (Fig. 3b,c). However, over-representation of Old Forest relative to Savannah communities also reduced acquisition costs in scenarios not including carbon value, indicating that Old Forest habitat was on average less valuable than Savannah habitat in the region, perhaps due to high human amenity values (Table 2; e.g., [58]). Scenarios maximizing β-diversity also met conservation targets by protecting less total area and up to an 11% cost savings compared to scenarios maximizing α-diversity (Fig.3b), and this finding was largely independent of carbon value or conservation target (Fig.3b). Prior studies of the consequence of emphasizing α- versus β-diversity in conservation planning have concluded that a focus on β-diversity is likely to enhance long term persistence in diverse species assemblages and reserve networks [22], [59], [60]. Our results broaden these conclusions by showing that scenarios that maximize β-diversity may also reduce the cost of conservation by reducing the area required to meet realistic targets for land acquisition.

### Conservation cost and carbon

Our results indicate that carbon credit sales should be considered as an option to maximize return on conservation investments in regions where land cost is high and old or growing forests offer habitat for valued focal communities. Our results therefore compliment suggestions that carbon credit sales have the potential to advance conservation and mitigate the impacts of climate change [13], [61], [62] but extend those suggestions by providing a spatially explicit, empirical example applied to a landscape with high conservation and cultural values [26], [58], [63]. The largest reductions in cost due to carbon credit sales were obtained by including carbon storage and carbon sequestration potential (Fig. 4), indicating that flexibility in carbon credit sales with respect to forest age can also increase economic efficiency. Although our results are based on a 20 year time-frame due to uncertainty about fire frequency, versus more typical 100 year time-frame for such projects [41], [61], they could easily be revised with new data.

Our finding that carbon storage reduced costs more than sequestered carbon (Fig. 2,4) is partly a consequence of logging history, given that close to 30% of the region not converted to exclusive human use is covered by forest ≥80 years-old. The predominance of young forest has the potential to reduce adjacency between older, high-value forest and savannah habitat with rich and diverse native bird communities. However, young forest patches may also provide relatively low-cost opportunities to link high-value patches where acquisition costs can be offset by relatively high sequestration rate. Nevertheless, most scenarios preferentially included older stands with more carbon storage, but lower sequestration rates (Fig. S4,S5 in Appendix S2). Several other studies have suggested that carbon credits could be used to advance conservation, particularly on private land to compensate land owners for forgone opportunity costs [18], [64], [65]. We extended these suggestions by providing a particularly detailed example to demonstrate how land use planners might offset the costs of conservation area design by acquiring habitats that simultaneously maximize the diversity of valued vertebrate communities and realize the economic potential of carbon credit sales.

As an alternative to the sale of carbon credits to reduce conservation costs, land conservation and protection on private lands, accomplished through relationship building and alternative tax plans, could be an option [7], [66], [67]. But, one of the biggest challenges to realizing a theoretical approach of implementing private land conservation agreements on high value biodiversity landscapes is the need to work with landowners willing to put conservation agreements on their land [7]. In Canada, and British Columbia in particular, there are some incentives in place such as tax credits ([9], e.g. Natural Area Protection Tax Exemption Program), but none of the currently implemented compensation schemes would compensate land owners for the lost opportunity costs of developing their land or using it in other revenue-generating manners such as via agriculture or forestry, as proposed elsewhere [68]–[70]. Currently, a private conservation agreement approach depends largely on individuals wanting to create a legacy and see their property protected into the future [71]. It was beyond the scope of our study to address issues related to a landowners motivation to participate in conservation [7], [72], nor did we want to speculate on the use of tax structure shifts.

### Conservation targets

A key goal of our work was to demonstrate novel routes to protecting high-value, Old Forest and Savannah bird communities at landscape scales in western North America. However, the amount of habitat needed to achieve those goals remains uncertain. Policy-driven targets for biodiversity conservation place goals for terrestrial habitat conservation at 17% by 2020 [73], but recent reviews suggest much higher targets (25–75%; [38]). We used a range of targets to explore their influence on reserve design, carbon value and the conservation of Old Forest and Savannah ecosystems, but we found that carbon contributed proportionally less to acquisition costs as targets increased in all scenarios (Fig.4) because higher targets required the acquisition of more expensive parcels. Thus, although total carbon generally increased linearly with conservation target, acquisition costs increased exponentially, causing a decline in relative benefit (Fig.4). However, even for the largest targets (50%) in α and β-diversity scenarios, carbon values reduced acquisition cost by 9.6 and 10.5%, respectively ($235, 227 M; Fig. 4) at $12.5 per Ton.

A number of uncertainties in our study also have the potential to limit its interpretation. First, actual purchase costs may differ from assessed or predicted values [7], [74]. Second, it may not be feasible to protect the areas offering the highest conservation value and least cost, particularly if regional representation or the augmentation of existing conservation areas is emphasized [75]. Third, although our results were robust over a range of carbon values, carbon markets remain unpredictable. Nevertheless, carbon markets are of substantial size, the European Union Emissions Trading System for example included 2.1 billion metric tons in 2011 [76]. In 2013 China, the largest national source of greenhouse gases (19.1% of total emissions), introduced pilot emission trading schemes [77], [78], joining a growing number of countries with national emission trading schemes [16]. Voluntary carbon markets that are currently the biggest market place for forest carbon offset projects in countries like Canada had a market volume of $572 M in 2011 [76]. Assuming that carbon markets develop further, our results demonstrate that carbon value has the potential to substantially reduce land acquisition costs in human-dominated landscapes, particularly in the Georgia Basin of western North America, where diverse Old Forest and Savannah bird [19] and plant [79] communities still persist in relatively isolated, mature forest and woodland habitats.

## Supporting Information

Appendix S1 Further details on our bird score modelling approach as well as residual spatial autocorrelation test results and community scores for individual bird species. We further present maps of the Old Forest, Savannah and β-scores.
(DOCX)

Appendix S2 Summary results of carbon storage and carbon sequestration potential modelling and a technical report on the FORECAST modelling approach used.
(DOCX)

Appendix S3 The R code we developed for Marxan calibration that can be used in combination with both the 32 and 64bit versions of Marxan. Function results can be used for Marxan post processing in R.
(DOCX)

Appendix S4 The expert elicitation introduction document that expert birders used as guidelines for their input.
(DOCX)
