# Supplementary material for: Bird Community Conservation and Carbon Offsets in Western North America
Source: PLoS One. 2014 Jun 11;9(6):e99292. doi: 10.1371/journal.pone.0099292 (PMC4053395; doi:10.1371/journal.pone.0099292)

**Appendix S1: Details of biodiversity modeling approach**

Following are the details of our biodiversity models as they have been presented in Schuster & Arcese [1], plus some modifications to reflect the changes we have made to our methodology since that paper was published.

**Study area and sampling methods**

We focused on a 2,520 km^2^ portion of the Coastal Douglas Fir zone of BC that includes >2000 islands from 0.0003 - 32,000 km^2^ (e.g. Vancouver Island). Roughly 40% of the CDF region still occurs as uneven-aged forest, interspersed with shallow soil balds, deep soil meadows and woodland/savannah habitat. Mature CDF forests support long-lived conifers and a mainly deciduous tree and shrub sub-canopy subject to disturbance and, as a result, are structurally complex [2,3]. As part of a larger program on avian conservation [4,5], we conducted point counts on 53 islands from 30 Apr – 11 Jul, 2005 - 2011 (except 2006). Trained observers recorded all birds detected in a 50m radius in a 10 min period between 5 AM – 12 PM, at 713 sample locations (mean distance between all locations = 19 km). Total visits to each location ranged from 1-12 (mean = 1.86), with each location recorded by handheld GPS (GPS60, Garmin Ltd, Kansas, USA).

**Expert rankings**

We asked 11 professional ornithologists with >5 years of experience with local birds to estimate the degree of association of 47 species (Table S1) to old forest (OF) and savannah/woodland (SAV) CDF habitats. Specifically, experts ranked species’ according to their expected association (low = -1, medium = 0 or highly associated = 1) with each of 10 focal habitat types in present-day CDF habitats using photographic and text descriptions of herbaceous, shrub, woodland, wetland, four forest types (pole, young, mature and old), and 2 human-dominated habitats (rural, urban). (For detailed descriptions of our approach see Appendix S4).

Expert ranks were then averaged for each species and habitat type. Old forest (OF) association scores for each species were then calculated by summing a species’ rank in each habitat, multiplied by following weights: herbaceous (-2), shrub/herb (-1), pole/sapling (-0.5), young forest (+0.5), mature forest (+1) and old forest (+2). Doing so resulted in a score for each species that ranged from a minimum of -7, indicating no association to old forest structure, to a maximum of 7, indicating a strong association to old forest structure. This score was then standardized to fall between -1 and 1 by dividing by the maximum value possible (7). All birds with positive forest association scores were therefore considered to be members of the CDF old forest (OF) bird community, with those species having higher forest association scores contributing most to composite maps. In this paper we extended this approach to also include Savannah habitats (SAV), which is detailed in the main text.

**Landscape covariates**

Because birds respond to many fine and coarse scale habitat features [6] we developed covariate descriptors of landscape condition and context using coarse (1km) and fine (100m) scale features to advance early work conducted only at coarse scales [7]. For modelling species detection and occurrence, we chose candidate predictors based on their proven ability to predict species occurrence at site and landscape levels in similar exercises or regions [4,8]. All covariate names appear in Table S2 and were derived from the following sources: (i) Terrain Resource Information Management (TRIM, http://archive.ilmb.gov.bc.ca/crgb/pba/trim/specs/specs20.pdf); (ii) Sensitive Ecosystems Inventory (SEI): East Vancouver Island and the Gulf Islands (http://www.env.gov.bc.ca/sei/); (iii) Earth Observation for Sustainable Development Landcover (EOSD LC 2000,[9]); (iv) aerial photographs to calculate the islands sizes; and (v) Terrestrial Ecosystem Mapping (TEM) of the CDF Zone [10].

Our dataset comprised 25 predictor covariates of site and landscape condition (Table S2), derived at each of 713 avian point count locations. The data source satellite and aerial photography imagery was collected between 2002 and 2004, which was in part supplemented by ground work until 2008. All covariates were created using Geospatial Modeling Environment [11] in conjunction with ArcGIS 10.1 [12] and R v. 2.15.2 [13]. Due to their widely varying scales, all covariates were standardized about their mean value, to ensure that importance was not driven by measurement scale [14].

**Occupancy and detection models**

The large size of our study area required us to compile data from related surveys conducted during a single 9 week period [4,5] but differing in sampling intensity between years and precluding reliable estimates of colonization and extinction [15]. We also assumed no variation in site occupancy across years to minimize model complexity, thus assumed a closed population for all species [16]. The R package unmarked v. 0.9-9 [17] provided the framework for all species models, which necessarily include two parts: occupancy and detection [16]. To estimate detectability we used one site specific (crown closure) and three observation specific (time of date, Julian date and observer identity) covariates. For each of 47 focal species we fitted all 16 detectability models (without parameterizing occupancy) and then ranked each by AIC [18] to select top-ranked models for further analysis. To accommodate our reduced but still extensive set of 25 predictor covariates for occupancy modelling, we first used a modified ‘stepwise’ covariate selection procedure linked to the unmarked package to create a candidate set of models based on the statistical significance of individual covariates and AIC. We then ranked all candidate models by AIC and averaged those with $\Delta$AIC ≤ 7 from the top ranked one [19].

**Predictive maps**

We created landscape level predictive occupancy maps over our 2,520 km^2^ study area using 252,000 1ha hexagons. For each hexagon centroid we generated a covariate set identical to that used for survey points, and then estimated probability of occurrence based on our averaged models for each of the focal species. To consolidate focal species maps into an index of forest-associated bird species richness, we created a score for each polygon resembling a single-species habitat suitability index [8,20]. Specifically, we calculated the polygon scores by summing the weighted probability of occurrence of each species linked to old forest structure via expert questionnaires [21]. This process yielded a weighted, forest-association community score that ranged from 0 (no forest-associated species present) to 1 (all forest-associated species present) for each of the map polygons.

**Additions to Schuster & Arcese** [1]

In addition to extending the extend of Schuster and Arcese (2013) we also addressed two uncertainties they identified to improve and assess the fit of our models by testing for spatial autocorrelation using Bayesian approaches to estimate Moran’s I in model residuals and goodness of fit using ‘area under the curve’ (AUC). Where spatial autocorrelation resulted in values of Moran’s I > 0.2, we added a spatial autocovariate to models. To assess model goodness of fit we used MCMC iteration to calculate AUC by comparing model results to BUGS estimates of latent occupancy state, using a modification of Zipkin et al. (2012).

The tests for spatial autocorrelation in model residuals resulted in Moran’s I > 0.2 in six of the 47 bird species. For five species (dark eyed junco, Eurpean starling, fox sparrow, northern rough-winged swallow, song sparrow) the inclusion of a first order autocovariate term resulted in a reduction of Moran’s I below 0.2. For one species (varied thrush) we did not find a first order autocovariate distance that removed autocorrelation entirely (Table S1). Bayesian model goodness of fit (GOF) estimates for AUC ranged from 0.659 – 0.991 (mean = 0.881) (Table S1). Based on the expert elicitation results we included 16 bird species in the OF score (Table S3, Figure S1) and 13 in the SAV score (Table S3, Figure S2). Five additional species had positive OF scores but were not included in the OF community score, because their 95% credible intervals of model GOF did not span AUC values of 0.8, which we used as our cut-off to indicate models of good fit (bald eagle, common raven, warbling vireo, Western tanager) or model predictions were unrealistic (Swainson’s thrush), predicting high probability of occurrence in urban areas, despite the fact that this species is mostly associated with undisturbed forests in BC [23]. The CDF score we created by combining OF (Fig. S1) and SAV (Fig. S2) predictions varied from 0 to 0.79, illustrating the fact that there is no area of complete overlap between the two biodiversity metrics, which was to be expected given the fact that we used fine scale (1ha) polygons as our predictive units (Figure S3).

References

1. Schuster R, Arcese P (2013) Using bird species community occurrence to prioritize forests for old growth restoration. Ecography (Cop) 36: 499–507. doi:10.1111/j.1600-0587.2012.07681.x.

2. Mosseler A, Thompson I, Pendrel BA (2003) Overview of old-growth forests in Canada from a science perspective. Environ Rev 11: S1–S7. doi:10.1139/a03-018.

3. Meidinger D, Pojar J (1991) Ecosystems of British Columbia. Victoria, BC: British Columbia Ministry of Forests.

4. Jewell KJ, Arcese P, Gergel S (2007) Robust predictions of species distribution: Spatial habitat models for a brood parasite. Biol Conserv 140: 259–272. Available: http://linkinghub.elsevier.com/retrieve/pii/S0006320707003291. Accessed 21 September 2010.

5. Martin TG, Arcese P, Scheerder N (2011) Browsing down our natural heritage: Deer impacts on vegetation structure and songbird populations across an island archipelago. Biol Conserv 144: 459–469.

6. Lawler JJ, Edwards TC (2006) A Variance-Decomposition Approach to Investigating Multiscale Habitat Associations. Condor 108: 47–58. Available: http://www.jstor.org/stable/4123195.

7. DeWan AA, Sullivan PJ, Lembo AJ, Smith CR, Maerz JC, et al. (2009) Using occupancy models of forest breeding birds to prioritize conservation planning. Biol Conserv 142: 982–991. Available: http://apps.isiknowledge.com/full_record.do?product=UA&search_mode=GeneralSearch&qid=1&SID=N2acHa91FCo5eMDPCkk&page=1&doc=1&colname=WOS. Accessed 30 October 2010.

8. Guisan A, Thuiller W (2005) Predicting species distribution: offering more than simple habitat models. Ecol Lett 8: 993–1009. Available: http://dx.doi.org/10.1111/j.1461-0248.2005.00792.x.

9. Wulder MA, White JC, Cranny M, Hall RJ, Luther JE, et al. (2008) Monitoring Canada’s forests. Part 1: Completion of the EOSD land cover project. Can J Remote Sens 34: 549–562. Available: http://apps.isiknowledge.com/full_record.do?product=UA&search_mode=OneClickSearch&qid=5&SID=1AJDGonKKCaodEhOhI4&page=4&doc=37&colname=WOS. Accessed 22 October 2010.

10. MES (2008) Terrestrial Ecosystem Mapping of the Coastal Douglas-Fir Biogeoclimatic Zone. Mandrone Environmental Services LTD., Duncan, BC. Available: http://a100.gov.bc.ca/pub/acat/public/viewReport.do?reportId=15273.

11. Beyer HL (2012) Geospatial Modelling Environment (Version 0.7.2.1). (software). URL: http://www.spatialecology.com/gme.

12. ESRI (2012) ArcGIS 10.1 Economic and Social Reserach Institute Inc., Redlands, CA. http://www.esri.com/.

13. R Development Core Team (2012) R: A language and environment for statistical computing 2.15.2, http://www.r-project.org.

14. White GC, Burnham KP (1999) Program MARK: survival estimation from populations of marked animals. Bird Study 46: 120–139. Available: http://www.informaworld.com/10.1080/00063659909477239.

15. MacKenzie DI, Nichols JD, Hines JE, Knutson MG, Franklin AB (2003) Estimating Site Occupancy, Colonization, and Local Extinction When a Species Is Detected Imperfectly. Ecology 84: 2200–2207. Available: http://www.esajournals.org/doi/abs/10.1890/02-3090.

16. Mackenzie DI, Nichols JD, Lachman GB, Droege SJ, Royle JA, et al. (2002) Estimating site occupancy rates when detection probabilities are less than one. Ecology 83: 2248–2255.

17. Fiske IJ, Chandler RB (2011) unmarked : An R Package for Fitting Hierarchical Models of Wildlife Occurrence and Abundance. J Stat Softw 43: 128–129. doi:10.1002/wics.10.

18. Akaike H (1974) A new look at the statistical model identification. IEEE Trans Automat Contr 19: 716–723. Available: http://ieeexplore.ieee.org/lpdocs/epic03/wrapper.htm?arnumber=1100705.

19. Burnham KP, Anderson DR (2002) Model selection and multimodel inference: a practical information-theoretic approach. New York, NY: Springer Verlag. Available: http://books.google.ca/books?hl=en&lr=&id=BQYR6js0CC8C&oi=fnd&pg=PR7&dq=burnham+and+anderson&ots=i8aZnjk7VG&sig=AEryxYBUSpBKb9_Vwz9DmwogkQ4. Accessed 21 October 2010.

20. Beaudry F, Pidgeon AM, Radeloff VC, Howe RW, Mladenoff DJ, et al. (2010) Modeling regional-scale habitat of forest birds when land management guidelines are needed but information is limited. Biol Conserv 143: 1759–1769. Available: http://www.sciencedirect.com/science/article/B6V5X-500SRHV-3/2/da232efc0ac0d36a999d1eb5c66493fb.

21. Martin TG, Kuhnert PM, Mengersen K, Possingham HP (2005) The power of expert opinion in ecological models using Bayesian methods: impact of grazing on birds. Ecol Appl 15: 266–280. Available: http://www.esajournals.org/doi/pdf/10.1890/03-5400. Accessed 18 October 2010.

22. Zipkin EF, Grant EHC, Fagan WF (2012) Evaluating the predictive abilities of community occupancy models using AUC while accounting for imperfect detection. Ecol Appl a Publ Ecol Soc Am 22: 1962–1972. Available: http://www.ncbi.nlm.nih.gov/pubmed/23210312.

23. Campbell RW, Dawe NK, McTaggart-Cowan I, Cooper JM, Kaiser GW, et al. (1997) The Birds of British Columbia: Passerines (Flycatchers Through Vireos). UBC Press.

Table S1: Bird species and elicitation results. Yellow fields show species that have positive OF scores, but have not been included in the combined metric, due to low GOF or unrealistic predictions (reasons in text). OF score = old forest association score. SAV score = savannah association score. OF/SAV weight = the weight positive OF/SAV scores got in producing the old forest/savannah community association metrics.

| **Bird Species** | **OF score** | **OF weight** | **SAV score** | **SAV weight** |
| --- | --- | --- | --- | --- |
| American Goldfinch | -0.396 | - | 0.236 | 0.092 |
| American Robin | -0.143 | - | 0.255 | 0.099 |
| Bald Eagle | 0.617 | - | -0.782 | - |
| Barn Swallow | -0.429 | - | -0.182 | - |
| Bewick's Wren | -0.221 | - | 0.055 | 0.021 |
| Brown-headed Cowbird | -0.532 | - | 0.400 | 0.156 |
| Brown Creeper | 0.831 | 0.077 | -0.600 | - |
| Chestnut-backed Chickadee | 0.636 | 0.059 | -0.255 | - |
| Chipping Sparrow | -0.487 | - | 0.255 | 0.099 |
| Common Raven | 0.682 | - | -0.673 | - |
| Dark-eyed Junco | -0.144 | - | 0.327 | 0.128 |
| European Starling | -0.331 | - | -0.218 | - |
| Fox Sparrow | -0.249 | - | -0.271 | - |
| Golden-crowned Kinglet | 0.734 | 0.068 | -0.636 | - |
| Hairy Woodpecker | 0.818 | 0.075 | -0.673 | - |
| Hammond's Flycatcher | 0.685 | 0.063 | -0.727 | - |
| House Finch | -0.275 | - | -0.076 | - |
| House Sparrow | -0.169 | - | -0.691 | - |
| House Wren | -0.092 | - | 0.051 | 0.020 |
| MacGillivray's Warbler | -0.123 | - | -0.109 | - |
| Northwestern Crow | -0.212 | - | -0.109 | - |
| Northern Flicker | 0.481 | 0.044 | -0.073 | - |
| Northern Rough-winged Swallow | -0.325 | - | -0.323 | - |
| Orange-crowned Warbler | -0.126 | - | 0.036 | 0.014 |
| Olive-sided Flycatcher | 0.539 | 0.050 | -0.455 | - |
| Pine Siskin | 0.578 | 0.053 | -0.382 | - |
| Pileated Woodpecker | 0.877 | 0.081 | -0.673 | - |
| Pacific-slope Flycatcher | 0.787 | 0.072 | -0.491 | - |
| Purple Finch | 0.235 | 0.022 | -0.182 | - |
| Red-breasted Nuthatch | 0.831 | 0.077 | -0.400 | - |
| Rufous Hummingbird | -0.175 | - | 0.364 | 0.142 |
| Red-winged Blackbird | -0.130 | - | -0.709 | - |
| Savannah Sparrow | -0.532 | - | 0.036 | 0.014 |
| Song Sparrow | -0.214 | - | 0.073 | 0.028 |
| Spotted Towhee | -0.184 | - | 0.164 | 0.064 |
| Swainson's Thrush | 0.429 | - | -0.345 | - |
| Townsend's Warbler | 0.760 | 0.070 | -0.655 | - |
| Tree Swallow | -0.186 | - | -0.364 | - |
| Varied Thrush | 0.770 | 0.071 | -0.764 | - |
| Violet-green Swallow | -0.200 | - | -0.200 | - |
| Warbling Vireo | 0.104 | - | -0.255 | - |
| White-crowned Sparrow | -0.552 | - | 0.309 | 0.121 |
| Western Tanager | 0.675 | - | -0.473 | - |
| Wilson's Warbler | -0.013 | - | -0.273 | - |
| Winter/Pacific Wren | 0.779 | 0.072 | -0.673 | - |
| Yellow-rumped Warbler | 0.412 | 0.038 | -0.436 | - |
| Yellow Warbler | -0.221 | - | -0.364 | - |

Table S2. Occupancy model covariate description including data source and covariate abbreviation used here.

| **Source** | **Covariate description** | **Abbreviation** |
| --- | --- | --- |
|  | Total amount of unpaved road length within a 1km buffer | rdl_up_1k |
|  | Total amount of unpaved road length within a 100 buffer | rdl_up_100 |
|  | Total amount of paved road length within a 1km buffer | rdl_p_1k |
| TRIM | Total amount of paved road length within a 100 buffer | rdl_p_100 |
|  | Nearest road | near_road |
|  | Nearest freshwater source | near_frshw |
|  | Nearest shoreline | near_saltw |
| Aerial photographs used to draw island polygons | Island size | Is_size |
|  | Rural/agriculture area within a 1km buffer | RUR_1KM |
| Terrestrial Ecosystem Mapping of the Coastal Douglas-Fir Biogeoclimatic Zone | Forest cover area within a 1km buffer including structural stages closed and young forest | FOR1_1KM |
|  | Forest cover area within a 1km buffer including structural stages mature and old forest | FOR2_1KM |
|  | Herbaceous area within a 1km buffer | HRB_1KM |
|  | Savannah area within a 1km buffer | SAV_1KM |
|  | Shrub area within a 1km buffer | SHR_1KM |
|  | Wetland area within a 1km buffer | WET_1KM |
|  | Urban/industrial area within a 100m buffer | URB_100 |
|  | Rural area within a 100m buffer | RUR_100 |
|  | Forest cover area within a 100 m buffer including structural stages closed and young forest | FOR1_100 |
|  | Forest cover area within a 100 m buffer including structural stages mature and old forest | FOR2_100 |
|  | Herbaceous area within a 100m buffer | HRB_100 |
|  | Savannah area within a 100m buffer | SAV_100 |
|  | Shrub area within a 100m buffer | SHR_100 |
|  | Wetland area within a 100m buffer | WET_100 |
|  | Distance to nearest Urban area | Near_Urb |
| Earth Observation for Sustainable Development(EOSD) Landcover | Crown closure within a 100m buffer, combining EOSD crow closure categories (dense, open and sparse) into one measure | CR_CL |

Table S3: Results of tests for residual spatial autocorrelation and area under the receiver operation curve (AUC). Columns on the left show initial results with maximum Moran’s I values from the MCMC approach. AUC mean and 95% credible interval values are further shown. For bird species with max. Moran’s I values > 0.2 autocovariates were added to averaged models to try to account for residual autocorrelation. The distance column shows which autocovariate distance was chosen per bird and columns to the right of this show the Moran’s I improvements as well as changes to AUC values.

| Bird species | | max Moran's I | AUC  mean | AUC  2.5 | AUC  97.5 | distance | max Moran's I | AUC  mean | AUC  2.5 | AUC  97.5 |
| --- | --- | --- | --- | --- | --- | --- | --- | --- | --- | --- |
| AMGO | 0.17 | | 0.92 | 0.80 | 0.96 |  |  |  |  |  |
| AMRO | 0.05 | | 0.98 | 0.97 | 0.99 |  |  |  |  |  |
| BAEA | 0.15 | | 0.73 | 0.69 | 0.76 |  |  |  |  |  |
| BARS | 0.10 | | 0.99 | 0.99 | 0.99 |  |  |  |  |  |
| BEWR | 0.11 | | 0.90 | 0.86 | 0.94 |  |  |  |  |  |
| BHCO | 0.10 | | 0.97 | 0.96 | 0.98 |  |  |  |  |  |
| BRCR | -0.10 | | 0.96 | 0.94 | 0.98 |  |  |  |  |  |
| CBCH | -0.17 | | 0.96 | 0.93 | 0.97 |  |  |  |  |  |
| CHSP | 0.09 | | 0.88 | 0.84 | 0.91 |  |  |  |  |  |
| CORA | 0.09 | | 0.66 | 0.58 | 0.75 |  |  |  |  |  |
| DEJU | 0.20 | | 0.91 | 0.88 | 0.93 | 250m | 0.09 | 0.92 | 0.90 | 0.94 |
| EUST | 0.26 | | 0.86 | 0.83 | 0.88 | 250m | 0.06 | 0.89 | 0.86 | 0.91 |
| FOSP | 0.25 | | 0.90 | 0.88 | 0.92 | 200m | 0.12 | 0.90 | 0.82 | 0.94 |
| GCKI | 0.11 | | 0.86 | 0.80 | 0.90 |  |  |  |  |  |
| HAFL | 0.16 | | 0.84 | 0.61 | 0.98 | 1km | 0.11 | 0.76 | 0.62 | 0.84 |
| HAWO | -0.08 | | 0.84 | 0.77 | 0.90 |  |  |  |  |  |
| HOFI | -0.09 | | 0.92 | 0.88 | 0.95 |  |  |  |  |  |
| HOSP | 0.09 | | 0.98 | 0.94 | 0.99 |  |  |  |  |  |
| HOWR | 0.10 | | 0.87 | 0.84 | 0.89 |  |  |  |  |  |
| MGWA | 0.19 | | 0.97 | 0.95 | 0.99 |  |  |  |  |  |
| NOCR | 0.12 | | 0.88 | 0.86 | 0.90 |  |  |  |  |  |
| NOFL | -0.09 | | 0.78 | 0.72 | 0.84 |  |  |  |  |  |
| NRWS | 0.21 | | 0.83 | 0.76 | 0.89 | 200m | 0.18 | 0.78 | 0.58 | 0.91 |
| OCWA | 0.07 | | 0.99 | 0.96 | 0.99 |  |  |  |  |  |
| OSFL | 0.08 | | 0.90 | 0.88 | 0.92 |  |  |  |  |  |
| PISI | 0.06 | | 0.96 | 0.69 | 0.99 |  |  |  |  |  |
| PIWO | 0.06 | | 0.82 | 0.73 | 0.90 |  |  |  |  |  |
| PSFL | 0.08 | | 0.99 | 0.98 | 0.99 |  |  |  |  |  |
| PUFI | 0.14 | | 0.99 | 0.95 | 0.99 |  |  |  |  |  |
| RBNU | -0.09 | | 0.96 | 0.87 | 0.99 |  |  |  |  |  |
| RUHU | 0.13 | | 0.86 | 0.82 | 0.88 |  |  |  |  |  |
| RWBL | 0.17 | | 0.99 | 0.98 | 0.99 |  |  |  |  |  |
| SAVS | 0.09 | | 0.95 | 0.86 | 0.98 |  |  |  |  |  |
| SOSP | 0.22 | | 0.80 | 0.81 | 0.85 | 250m | 0.09 | 0.85 | 0.82 | 0.87 |
| SPTO | 0.19 | | 0.92 | 0.91 | 0.94 |  |  |  |  |  |
| SWTH | 0.14 | | 0.79 | 0.74 | 0.84 |  |  |  |  |  |
| TOWA | 0.06 | | 0.97 | 0.95 | 0.98 |  |  |  |  |  |
| TRES | 0.12 | | 0.84 | 0.76 | 0.91 |  |  |  |  |  |
| VATH | 0.26 | | 0.79 | 0.68 | 0.88 | --- |  |  |  |  |
| VGSW | 0.04 | | 0.89 | 0.85 | 0.93 |  |  |  |  |  |
| WAVI | 0.16 | | 0.74 | 0.69 | 0.79 |  |  |  |  |  |
| WCSP | 0.13 | | 0.88 | 0.85 | 0.90 |  |  |  |  |  |
| WETA | 0.07 | | 0.66 | 0.56 | 0.77 |  |  |  |  |  |
| WIWA | 0.18 | | 0.88 | 0.84 | 0.92 |  |  |  |  |  |
| WIWR | 0.08 | | 0.81 | 0.77 | 0.84 |  |  |  |  |  |
| YRWA | 0.09 | | 0.79 | 0.74 | 0.84 |  |  |  |  |  |
| YWAR | 0.17 | | 0.85 | 0.81 | 0.88 |  |  |  |  |  |

Fig S1: Old Forest score reprentation.


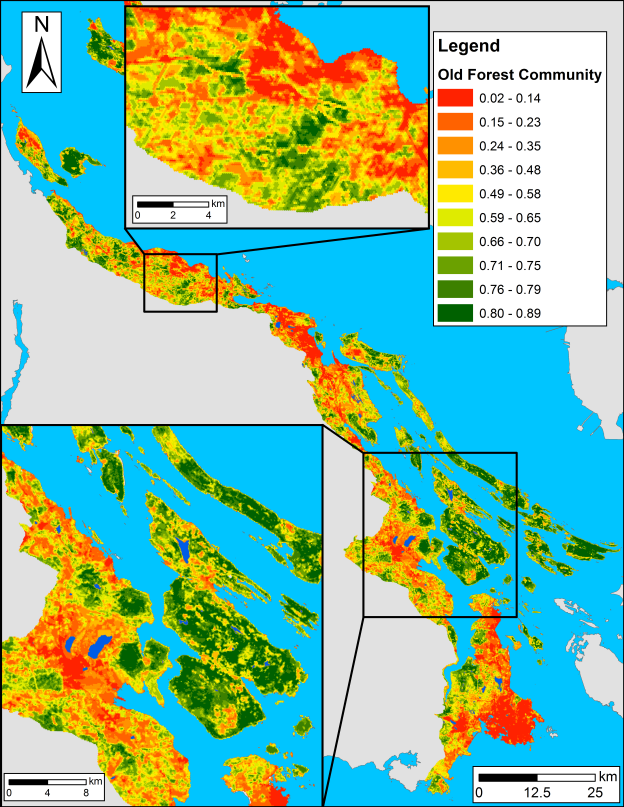


Fig. S2: Savannah score representation.


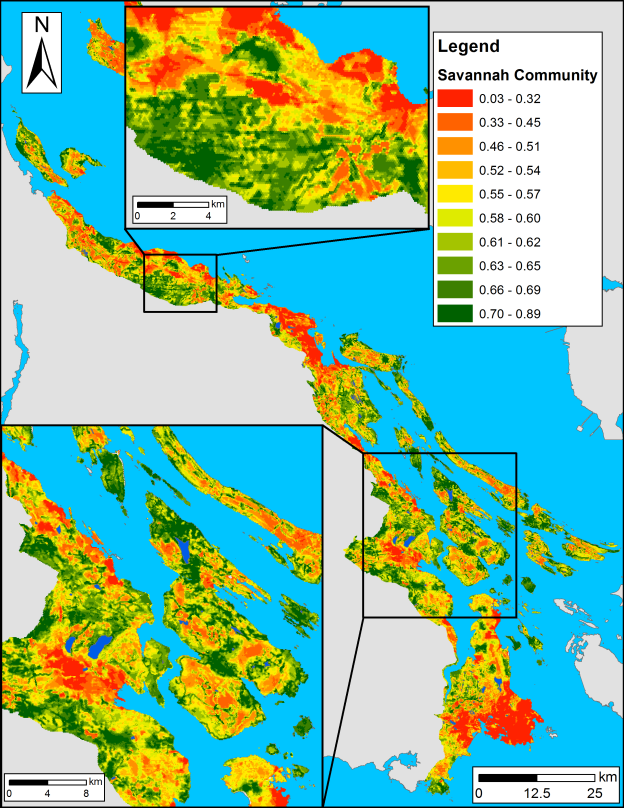


Fig. S3: Beta Score representation.


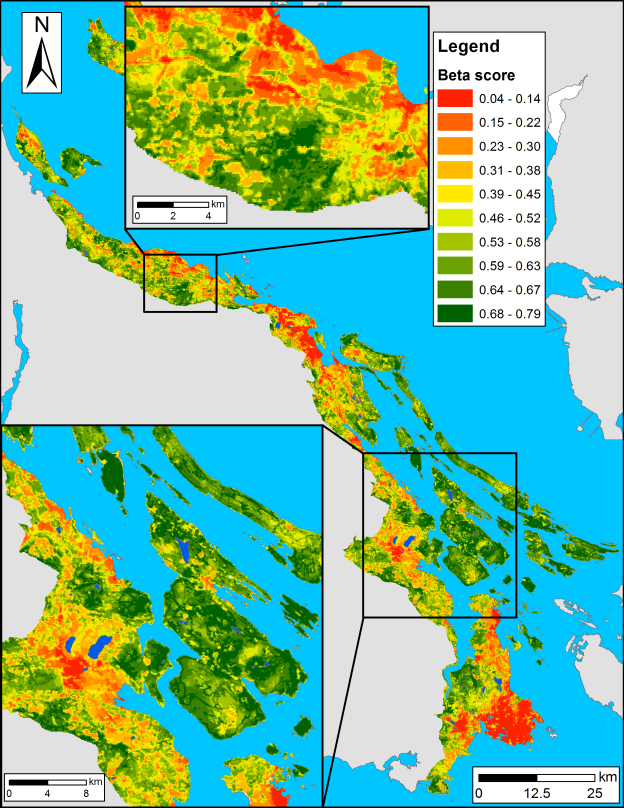

Supplement: Appendix S1 — Further details on our bird score modelling approach as well as residual spatial autocorrelation test results and community scores for individual bird species. We further present maps of the Old Forest, Savannah and β-scores. (DOCX) [file pone.0099292.s001.docx]
