# Supplementary material for: Bird Community Conservation and Carbon Offsets in Western North America
Source: PLoS One. 2014 Jun 11;9(6):e99292. doi: 10.1371/journal.pone.0099292 (PMC4053395; doi:10.1371/journal.pone.0099292)

**Appendix S2: Carbon model details**

Standing carbon estimates from model FORECAST in the CDF ranged from 0 to 615 t/ha (mean ± SD = 96.1 ± 92.95) and the total amount of carbon in the study area was estimated at 20.84 Mt (Figure S4). Estimates for sequestration potential over the next 20 years ranged from -9 to 130.6 t/ha (mean ± SD = 37.4 ± 31.9) with a total sequestration potential for the area of 7.98 Mt (Figure S5).

The methodology for standing carbon and carbon sequestration potential estimates is described in a technical report [1]. Terrestrial Ecosystem Mapping (TEM) data from Parks Canada CDF areas were combined with TEM data from non-Parks areas [2]. The merged TEM data were structured such that each polygon was divided into up to three discrete subsections. Each subsection (defined in the data as a proportion of the whole polygon area) had a site series and structural stage defined. Site series are representative ecosystems in each Biogeoclimatic subzone of British Columbia [2]. For the carbon modeling Seely [1] used FORECAST [3], a stand-level forest ecosystem simulator that is one of two models approved by the BC Ministry of Forests for carbon budget assessments [4], and the only model calibrated for use in the CDF [5] and linked to TEM [6].

To facilitate the carbon analysis, the TEM polygons were stratified into a series of relatively homogenous analysis units (AUs) based upon site series. The starting conditions for the FORECAST simulations were created by running the model in set-up mode up to 2000 years, to allow for the accumulation of dead organic matter (including soil organic matter) that would be representative of the disturbance history of the site. The final set-up run was conducted with nutrient feedback turned on to allow the site quality representation in the model to stabilize.

To represent the impact of endemic disturbance agents, including pests and pathogens, the simulations included periodic mortality events that occurred throughout the simulation at a regular interval and intensity for each species. Carbon storage curves (annual time step) were created for each AU and stored in a database. Carbon contents were simulated for all ecosystem pools including: soil organic matter, above-ground litter, above and below-ground tree biomass, plant biomass, deadwood, and dead below-ground biomass. Net ecosystem carbon storage was calculated for each AU considering the fact that only certain ecosystem pools are generally eligible for forest carbon offsets. Net ecosystem carbon storage was limited to: above and below-ground tree biomass, deadwood biomass, and dead below-ground biomass. Each AU was simulated for a period of 300 years with results reported for annual time steps.

FORECAST results were subsequently assigned to individual TEM polygons by estimating the age of each polygon subsection based upon the current assigned structural stage and estimated productivity class [6]. These age estimates are derived from ranges provided by Meidinger [7] for regional forest ecosystems. Ages of old stands (structural stage 7) were set at 200 to be conservative. Age estimates were verified against a subset of TEM polygons (Southern Gulf Islands) for which direct age estimates were available (n=254). For conservation prioritization analysis we used predicted net ecosystem carbon storage and net ecosystem carbon sequestration estimates for 20 years from now (the analysis timeframe predictions could be made with confidence).

References

1. Seely B (2012) Evaluation of Carbon Storage within Forests in the Coastal Douglas Fir Zone (unpiblished technical report): 14.

2. MES (2008) Terrestrial Ecosystem Mapping of the Coastal Douglas-Fir Biogeoclimatic Zone. Madrone Environmental Services LTD., Duncan, BC. Available: http://a100.gov.bc.ca/pub/acat/public/viewReport.do?reportId=15273.

3. Kimmins JP, Mailly D, Seely B (1999) Modelling forest ecosystem net primary production: the hybrid simulation approach used in FORECAST. Ecol Modell 122: 195–224.

4. Ministry of Environment BC (2011) Protocol for the Creation of Forest Carbon Offsets in British Columbia. British Columbia Ministry of Environment, Victoria. Available: http://www.env.gov.bc.ca/cas/mitigation/fcop.html.

5. Blanco JA, Seely B, Welham C, Kimmins JP (Hamish), Seebacher TM (2007) Testing the performance of a forest ecosystem model (FORECAST) against 29 years of field data in a Pseudotsuga menziesii plantation. Can J For Res 37: 1808–1820. Available: http://dx.doi.org/10.1139/X07-041.

6. Seely B, Nelson J, Wells R, Peter B, Meitner M, et al. (2004) The application of a hierarchical, decision-support system to evaluate multi-objective forest management strategies: a case study in northeastern British Columbia, Canada. For Ecol Manage 199: 283–305.

7. Meidinger D, Trowbridge R, Macadam A, Tolkamp C (1998) Field Manual for Describing Terrestrial Ecosystems. Victoria, Canada.: Crown Publications Inc.

Figure S4: Net ecosystem carbon storage within forests of the CDF zone in British Columbia.


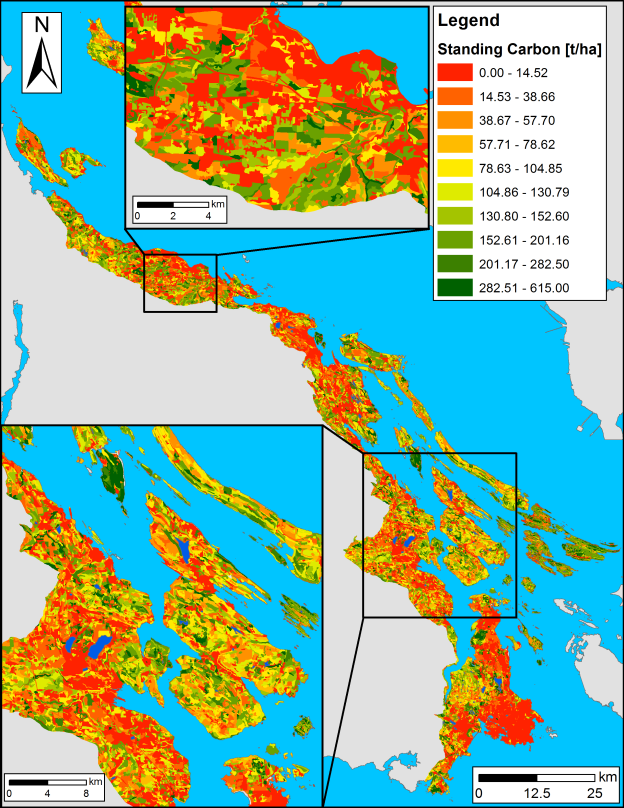


Figure S5: Net ecosystem carbon sequestration (during the next 20 years) within forests of the CDF zone in British Columbia.


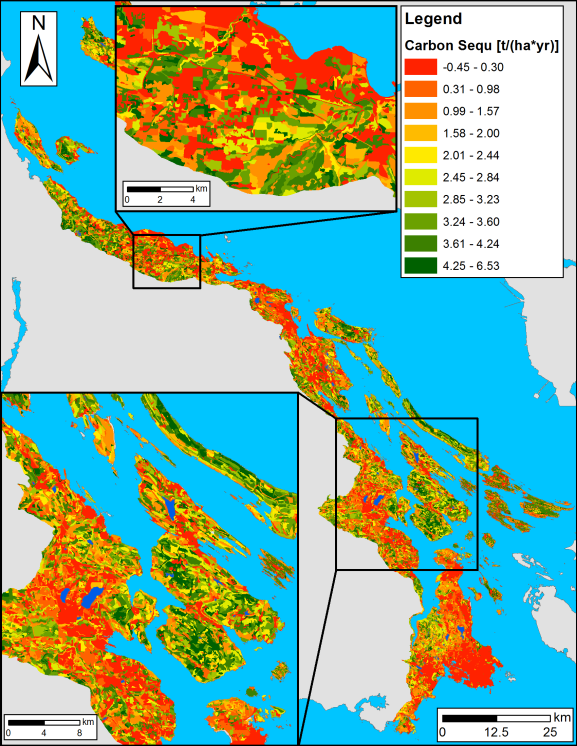

Supplement: Appendix S2 — Summary results of carbon storage and carbon sequestration potential modelling and a technical report on the FORECAST modelling approach used. (DOCX) [file pone.0099292.s002.docx]
