# Supplementary material for: Bird Community Conservation and Carbon Offsets in Western North America
Source: PLoS One. 2014 Jun 11;9(6):e99292. doi: 10.1371/journal.pone.0099292 (PMC4053395; doi:10.1371/journal.pone.0099292)
Supplement: Appendix S3 — The R code we developed for Marxan calibration that can be used in combination with both the 32 and 64bit versions of Marxan. Function results can be used for Marxan post processing in R. (DOCX) [file pone.0099292.s003.docx]

**Appendix S3: R function for Marxan batch run**

#bare bone run with standard input files

mm <- marxan()

#batch run with SPF, BLM, NREPS, NITNS changing

spf <- c(1,5,10,15,20)

blm <- c(0, 0.1, 0.2, 0.5, 1)

nreps <- c(10, 20, 50, 100)

nitns <- c(10000, 100000, 1000000, 10000000)

mm <- marxan(pu="pu_non_st.csv",

puvsp="puvsp_non_st.csv",

spec="spec_non_st.csv",

bound="bound_non_st.csv",

spf=spf, blm=blm, nreps=nreps, nitns=nitns, scenname="Full")

##################################################################

##################################################################

## R script for Marxan batch run

## returns data.frames of the best solutions, summed solutions,

## and the summary tables

##

## Parts based on code by Paulo Cardoso

## (http://lists.science.uq.edu.au/pipermail/marxan/2008-May/000319.html)

##

## Author: Richard Schuster (mail@richard-schuster.com

## 11 July 2013

##################################################################

##################################################################

marxan <- function(pu="pu.dat", puvsp="puvsp2.dat", spec="spec.dat", bound="", spf=1,

blm=0, nitns=100000, nreps=10, scenname="", indir=getwd(), outdir=getwd() ){

## Read and obtain Input.dat parameters

if(file.exists("input.dat"))

input.file<-dir(pattern="input.dat")

else

stop("input.dat file not found")

input<-readLines(input.file[1],n=-1)

## Set input and output directories

txt <- "INPUTDIR"

input[grep(txt, input)]<-paste(txt,sprintf("%s/input",indir))

txt <- "OUTPUTDIR"

input[grep(txt, input)]<-paste(txt,sprintf("%s/output",outdir))

## Create data frame for later use with GIS

txt <- "PUNAME"

input[grep(txt, input)]<-paste("PUNAME",pu) # pu file

txt <- "PUVSPRNAME"

input[grep(txt, input)]<-paste(txt,puvsp) # Puvsp file name in input.dat

spffr <- read.csv((file=sprintf("%s/input/%s",indir,spec)))

txt <- "SPECNAME"

input[grep(txt, input)]<-paste(txt,spec) # Spec file name in input.dat

#Boundary file

txt <- "BOUNDNAME"

input[grep(txt, input)]<-paste(txt,bound) # pu file

#gsub(" ","",substr(input[grep(txt, input)],nchar(txt)+1,nchar(input[grep(txt, input)])), fixed=T)

pufr <- read.csv((file=sprintf("%s/input/%s",indir,pu)))

ssolnfr <- bestfr <- data.frame(ID= pufr$id)

summed <- list(TimeStamp=date())

####################### Step 2

##################################################################

##Loop for sequential sfp

kk <- 2

for(ii in 1:length(spf)) {

#species penalty factor needs tp be set and saved win spec file for each run

spffr$spf <- spf[ii]

write.csv(spffr, sprintf("%s/input/%s",indir,spec), row.names = FALSE)

## Loop for sequential Marxan Runs

for(jj in 1:length(blm)){

## Input.dat parameters ##

txt <- "BLM"

input[grep(txt, input)]<-paste(txt,blm[jj]) # BLM in input.dat

for (ll in 1:length(nreps)){

txt <- "NUMREPS"

input[grep(txt, input)]<-paste(txt,nreps[ll]) # Number of runs in input.dat

for (mm in 1:length(nitns)){

txt <- "NUMITNS"

input[grep(txt, input)]<-paste(txt,sprintf("%i",nitns[mm])) # Number of runs in input.dat

txt <- "SCENNAME"

runname <- sprintf("%s_Spf%i_Blm%s_Nrep%i_Iter%i",scenname,round(spf[ii])

,blm[jj],nreps[ll], nitns[mm])

input[grep(txt, input)]<-paste(txt,runname) # Puvsp file name in input.dat

write(input,"input.dat")# Re-write input file at each run with the

#corresponding parameters changed

if(file.exists("Marxan_x64.exe"))

system("Marxan_x64.exe",wait=T,invisible=T) # Call Marxan to execute

else if(file.exists("Marxan.exe"))

system("Marxan.exe",wait=T,invisible=T) # Call Marxan to execute

else

stop('No Marxan executable found in working directory')

# saving results for function return

ssoln <- read.csv((file=sprintf("%s/output/%s_ssoln.txt",outdir,runname)))

best <- read.csv((file=sprintf("%s/output/%s_best.txt",outdir,runname)))

summ <- read.csv((file=sprintf("%s/output/%s_sum.txt",outdir,runname)))

ssoln<-ssoln[order(ssoln$planning_unit),]

best<-best[order(best$planning_unit),]

ssolnfr <- data.frame(ssolnfr, ssoln$number)

bestfr <- data.frame(bestfr, best$solution)

summed[[kk]] <- summ

names(ssolnfr)[kk] <- names(bestfr)[kk] <- names(summed)[kk] <- runname

kk <- kk + 1

}

}

}

}

return(list(ssoln=ssolnfr,best=bestfr,sums=summed))

}

###################################################################

#### FUNCTION END

###################################################################
