# Supplementary material for: Bird Community Conservation and Carbon Offsets in Western North America
Source: PLoS One. 2014 Jun 11;9(6):e99292. doi: 10.1371/journal.pone.0099292 (PMC4053395; doi:10.1371/journal.pone.0099292)
Supplement: Appendix S4 — The expert elicitation introduction document that expert birders used as guidelines for their input. (DOCX) [file pone.0099292.s004.docx]

**1. Introduction**


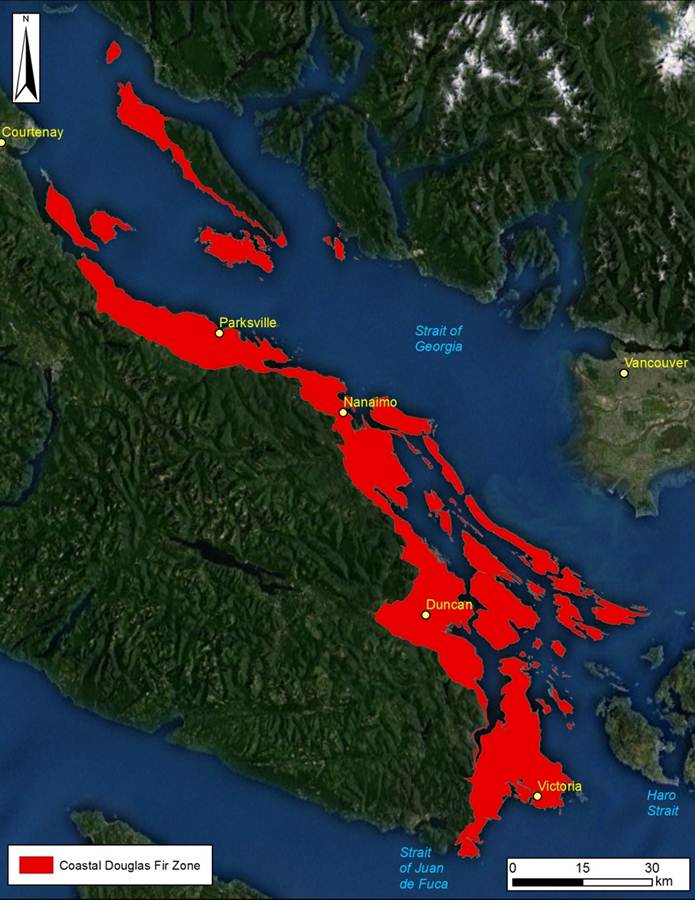
Thank you for taking time to complete our survey, which we believe will take 30-50 minutes. We hope the knowledge that you are helping to identify high quality terrestrial habitats for conservation in the Coastal Douglas Fir Zone (CDF, see figure) and our offer of a $100 honorarium makes your effort worthwhile.

We are mapping bird communities of interest to conservation in the CDF using 47 bird species maps based on thousands of `point counts` and expert opinion to associate species with habitat type and condition. Once assembled, these and other maps of native and exotic plant species will be used to identify old forest, woodland and wetland habitats within the CDF that are particularly likely to contribute positively to the persistence of diverse native bird and plant communities in future. Our finished maps will be available to land use planners and facilitate ongoing reserve design exercises aimed at maximizing the persistence of native species and ecosystems of the CDF Zone by identifying and conserving priority sites.

We are requesting your help because detailed information on the reliance of birds on the habitats occurring in the Coastal Douglas fir zone are lacking for most species, but well-established in the collective experience of many dedicated birders and biologists. We are therefore reaching out to experienced birders to help us summarize this local knowledge by filling out the attached a survey. In return, we promise to gratefully acknowledge your help in all reports and publications we produce, to provide you with copies of those reports, and to send a $100 honorarium to the address you provide.

The accompanying Excel file asks you to rank bird species in terms of their broad ‘reliance’ (low, medium, high, or unknown) on the 10 habitat types of interest in the CDF zone. Although you will sometimes be uncertain about the correct response, we ask that you select the one that best represents your own experience in the region. At the end of the survey, please provide your contact information in the space provided so we can send you a honorarium.

Thank you very much for your help,

Richard Schuster and Peter Arcese

Centre for Applied Conservation Research
University of British Columbia
#3041 – 2424 Main Mall
Vancouver, BC V6T 1Z4

Email: [ubc@richard-schuster.com](mailto:ubc@richard-schuster.com) and [peter.arcese@ubc.ca](mailto:peter.arcese@ubc.ca)

**2. Habitat Reliance in Salish Sea Birds**

Please read the following descriptions of habitats found in the CDF Biogeoclimatic Zone, and then indicate the degree to which you expect that each listed bird species ‘relies’ or ‘uses’ each habitat type during the spring and summer breeding period (~Apr-Jul) using the embedded, drop-down menus in the accompanying Excel file. To keep acquainted with the habitats described, we have also attached a 1-page ‘key’ which you may wish to print and have at your side as you complete the spreadsheet.

| **Herbaceous:** Early successional or herbaceous communities maintained by environment or disturbance (e.g., flooding, grazing, fire, agriculture); dominated by forbs, graminoids, ferns. Invading or residual shrubs and trees may be present (tree cover < 10%, shrubs < 20%), time since disturbance < 20 years via forest succession or non-forested communities maintained in this stage. | 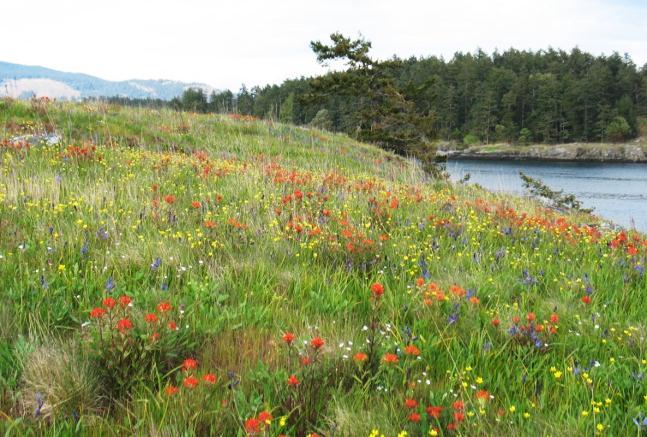  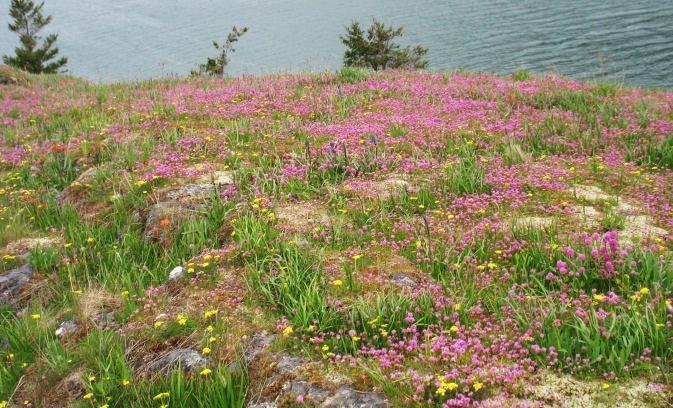 |
| --- | --- |
| **Shrub/Herb:** Early successional or shrub communities maintained by environment or disturbance; dominated by shrubby vegetation that is <10m tall. Seedlings and advance regeneration may be abundant (tree cover < 10%, shrub cover > 20%), time since disturbance < 40 years via forest succession. | 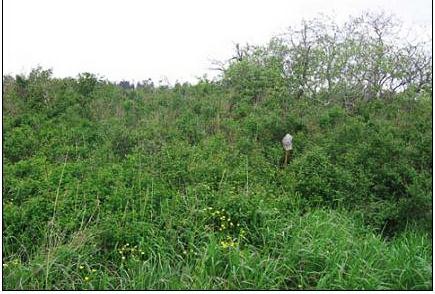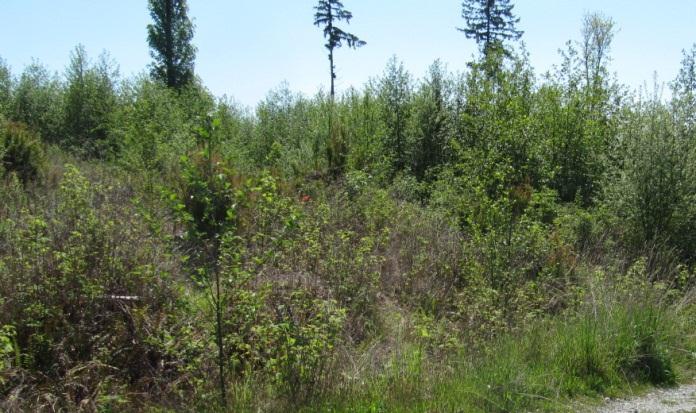 |
| **Pole/Sapling:** Trees > 10 m tall, typically densely stocked, have overtopped shrub and herbaceous layers; younger stands are vigorous (usually > 10-15 years old); older stagnated stands (up to 100 years old) are also included. Self-thinning and vertical structure not yet evident in the canopy, time since disturbance < 40 years via forest succession and up to 100+ years for dense stagnant stands. | 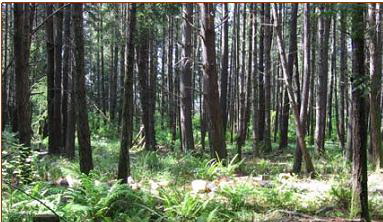 |
| **Young Forest:** Self-thinning has become evident and the forest canopy has begun to differentiate into distinct layers (dominant, main canopy, and overtopped); vigorous growth and a more open stand than in the Pole/Sapling stage, time since disturbance generally 40-80 years, depending on tree species and ecological conditions. | 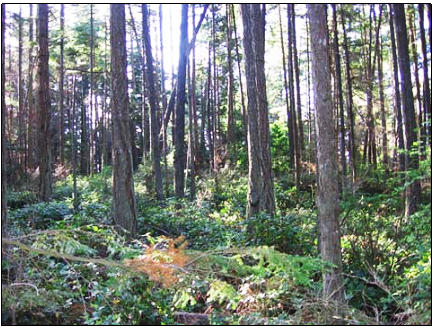 |
| **Mature Forest:** Trees established after the last disturbance have matured; a second cycle of shade-tolerant trees may have become established; understories become well developed as the canopy opens up; time since disturbance generally 80-250 years. | 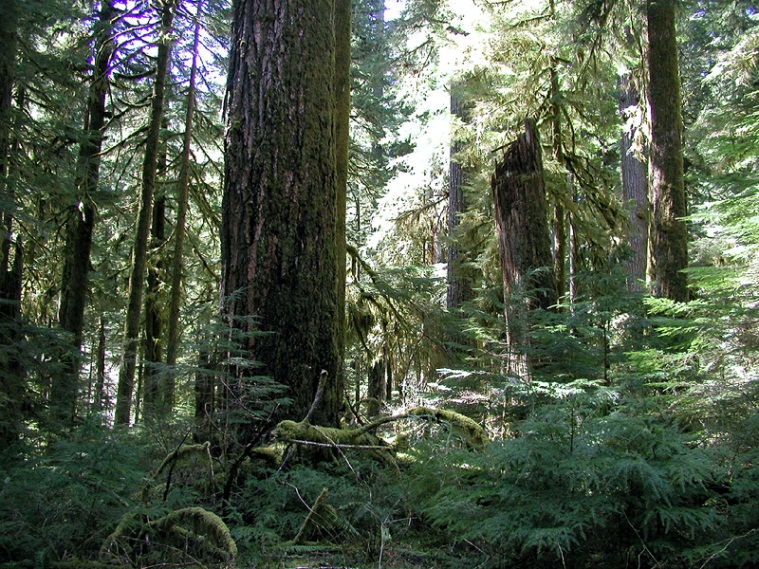 |

| **Mature Forest (cont.)** | 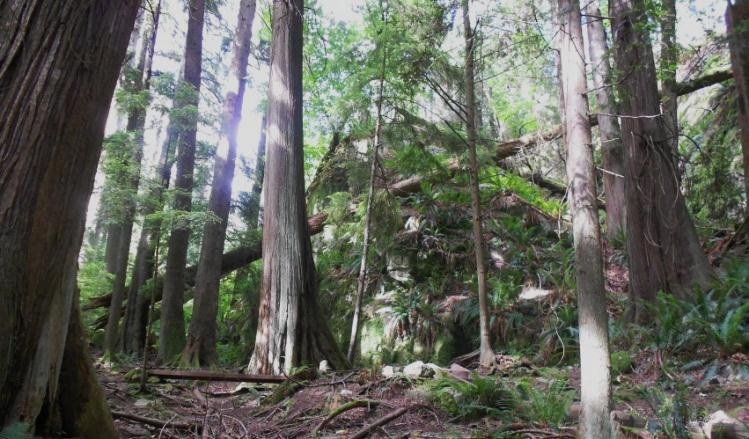 |
| --- | --- |
| **Old Forest:** Older, structurally complex stands comprised shade-tolerant and regenerating tree species, but often including long-lived, older seral stage trees sometimes dominating the upper canopy. Snags and coarse woody debris in all stages of decomposition, understory of deciduous and regenerating confers typical. Shrub layer well-developed and dense, especially in light gaps. Time since last stand-replacing disturbance generally > 250 years. | 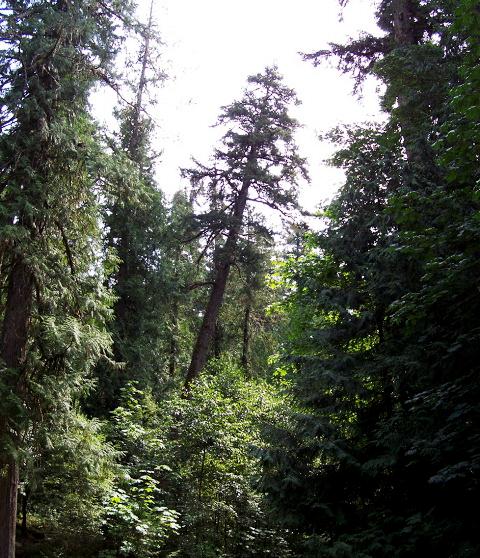 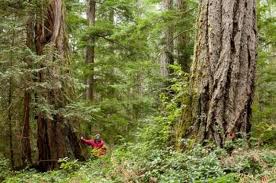 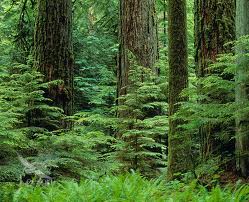 |

| **Rural/Agriculture:** Rural areas are characterize by areas that have residences and other human development scattered and intermingled with forests, range, farm land, cultivated fields or native vegetation. Cultivated fields are non-forested, open areas that are subject to agricultural practices including plowing, fertilization, and non-native crop production which often results in long-term soil and vegetation changes. Although cultivated fields are typically agriculture based our definition includes other green spaces as well, including city parks, baseball fields, residential lawns, and golf courses. | 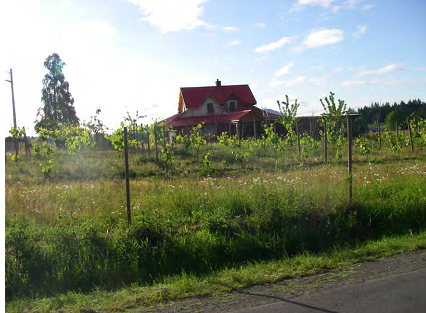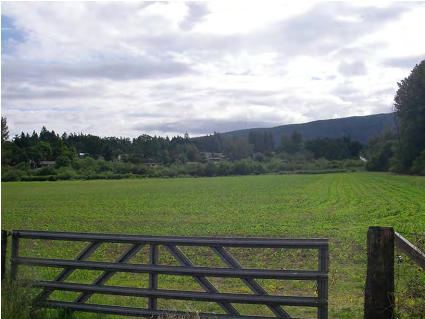 |
| --- | --- |
| **Urban/Industrial:** Urban units are characterized by an almost continuous covering over the landscape by residences and human development. Industrial areas dominated by industrial development namely, pulp and paper, lumber mills, oil/gas refineries and so on. These units are characterized by a high degree of ground disturbance; concrete parking lots, large commercial buildings, work yards and other specialized industry infrastructure.  Other urban/industrial areas include exposed soils (predominantly disturbance sites associated with new housing developments), Gravel pits, Mines, Railroad and Road surfaces. | 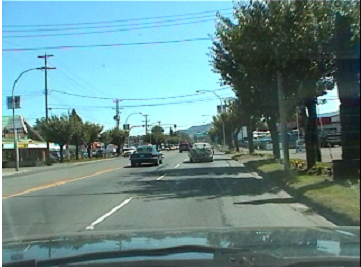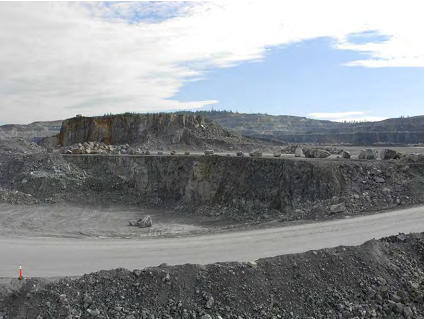 |

| **Woodland:** coniferous and mixed woodland (≤35% canopy; typically of Douglas fir, arbutus and oak), including and often inter-mixed with herbaceous and maritime meadow or shallow-soil, sparsely vegetated openings (herb-dominated ecosystems of southeastern Vancouver Island and many islands in the Strait of Georgia). In maritime and shallow soil GOE meadows, tree species are mostly or completely absent, but may include sparse cover of e.g., Garry Oak, Douglas-fir and shore pine, and shrubs in sparse to patchy accumulations.  For several reasons, we must exclude from our consideration ‘deep soil’ GOE meadows that would be expected to succeed relatively rapidly to continuous coniferous forest in the absence of disturbance sufficient to maintain them. | 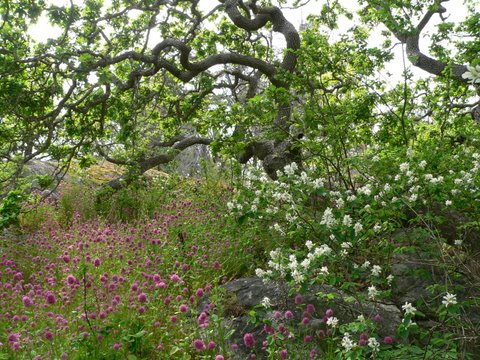  **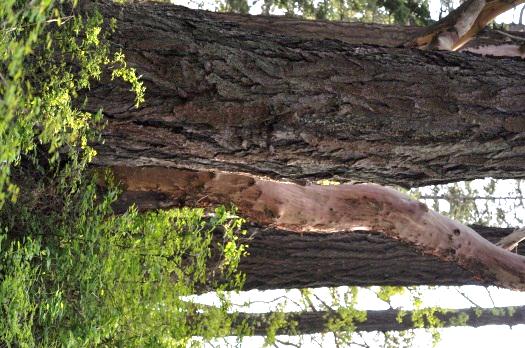** |
| --- | --- |
| **Wetland:** Wetlands are characterized with a water table at, near, or above the surface, daily, seasonally or year-round. Soils in wetlands are water-saturated for enough periods that excess water and low soil oxygen concentrations create conditions necessary for water-tolerant plants to dominate. This unit includes floodplains, fens, swamps, marshes, bogs, shallow open water and estuaries. | 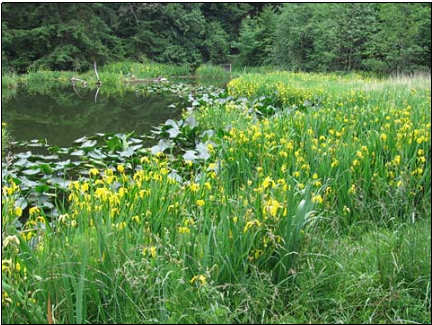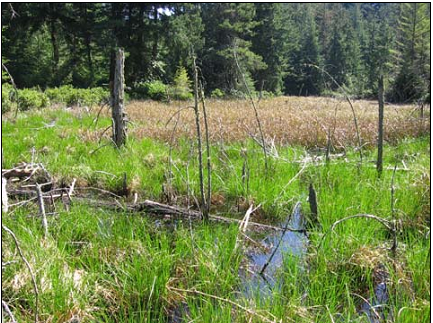 |
